# Technological characteristics of inulin enriched gluten‐free bread: Effect of acorn flour replacement and fermentation type

**DOI:** 10.1002/fsn3.2567

**Published:** 2021-09-06

**Authors:** Ameneh Shiri, Mohammad Hassan Ehrampoush, Seyed Ali Yasini Ardakani, Farimah Shamsi, Neda Mollakhalili‐Meybodi

**Affiliations:** ^1^ Department of Food Science and Technology School of Public Health Shahid Sadoughi University of Medical Sciences Yazd Iran; ^2^ Department of Environmental Health Engineering Environmental Science and Technology Research Center School of Public Health Shahid Sadoughi University of Medical Sciences Yazd Iran; ^3^ Department of Food Science and Technology Islamic Azad University Yazd Iran; ^4^ Department Biostatistics School of Public Health Shahid sadoughi University of Medical Sciences Yazd Iran; ^5^ Research Center for Food Hygiene and Safety Shahid Sadoughi University of Medical Sciences Yazd Iran

**Keywords:** acorn flour, fermentation, gluten‐free bread, inulin, rice flour

## Abstract

Textural, physicochemical, and sensory characteristics of rice‐based gluten‐free bread in the presence of acorn flour; inulin and different fermentation type (yeast starter fermentation [Y] or mixed fermentation based on sourdough [MF‐SD]) were investigated. Acorn flour was added to replace rice flour at a proportion of 10, 30, and 50% W/W. Furthermore, the mixture flour was replaced by inulin as a functional prebiotic ingredient at 10% W/W. Considering results obtained at this study, using mixed fermentation based on sourdough and inulin at 10% W/W provide the structure able to restore gases through baking process at formulations containing acorn flour at 30% W/W (A_30_R_70_SL). The highest specific volume (1.47 ± 0.04 cm^3^ g^−1^) and the lowest hardness (40.97 ± 0.87 N) are observed in A_30_R_70_SL which seems to be induced by its potential to form gel. Acorn flour substitution level at 50% W/W adversely influenced the technological characteristics of final product and its perception by the consumer. Acorn flour substitution up to 30% W/W is preferred by the consumer which is attributed to its potential role to improve the unpleasant pale color of rice‐based gluten‐free products. A negatively significant correlation has been observed between the color perception by the consumer and crumb lightness (*r* = −.493, *p* ≤ .05).

## Graphical abstract

The influence of fermentation type and acorn flour substitution level on technological characteristics of rice‐based gluten‐free bread in the presence 10% W/W long chain inulin has been investigated. Acorn flour is influencive in improving the technological characteristics and consumer perception of gluten‐free bread. The optimum formulation from technological perspective is found at samples containing 10% W/W inulin and 30% W/W acorn flour and fermented by mixed fermentation based on sourdough. Exopolysaccharides produced by mixed fermentation based on sourdough facilitate the structure formation.

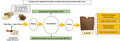

## 1 INTRODUCTION

Bread is staple foodstuff made and consumed in most countries around the world through its ease of use in an affordable cost (Mohammadi et al., 2021; Vatankhah et al., 2017). However, wheat flour is mainly used in making bread through its unique viscoelastic characteristics and textural behavior (Beltrão Martins, Gouvinhas, et al., 2020; Beltrão Martins, Nunes, et al., 2020; Meybodi et al., 2019), and its consumption is restricted in people suffering from celiac disease and other gluten‐related disorders (Mollakhalili Meybodi et al., 2015; Omedi et al., 2019).

Celiac disease (CD) with the global prevalence ratio of 1% is an autoimmune response in genetically susceptible persons exposed to certain oligopeptides sequence found in prolamine proteins like gliadin in gluten protein (Nejad et al., 2011; Wang et al., 2017). People who suffer from CD should follow a severe lifelong gluten‐free diet (Wang et al., 2017). Considering the unique characteristics of gluten protein, the desired characteristics of gluten‐free bakery products are difficult to be achieved compared to their gluten containing counterparts (Nejad et al., 2011; Omedi et al., 2019).

Different gluten‐free flours like rice, corn, sorghum, soy, and acorn can be inherently used in making gluten‐free bakery products (Rai et al., 2018) with the preference of using a mixture of two or three to increase the nutritional value and provide the desired textural and sensory characteristics (Arendt et al., 2008; Nikmaram et al., 2015). Rice flour is generally used as the basis flour regarding its tasteless, colorless, and hypoallergenic nature and being a rich source of sodium, fat, dietary fiber, and easily digested carbohydrates (Demirkesen et al., 2010a; Kadan et al., 2001). A considerable attention has been devoted to acorn as the fruit of *Quercus* genus tree regarding its high quantity of vitamins B, E, potassium, phosphorus, magnesium, iron, dietary fiber, essential fatty acids, and amino acids to complement the nutritional quality of rice flour‐based gluten‐free products (Demirkesen et al., 2010b; Sacchetti et al., 2004). It has also been stated that using acorn flour can improve the functional properties, color, and flavor of gluten‐free bakery products, depending on its level of use (Demirkesen et al., 2010b).

Considering the importance of gluten protein in preserving the gas produced during the fermentation process and providing the desired textural and structural characteristics of bread, the main technological deficiency of gluten‐free breads is their poor structure (Biesiekierski, 2017; Moore et al., 2006). Using inulin‐type fructans (ITFs) can improve the gas retention capacity by providing gel‐like structure and increasing the hydration ratio (Capriles & Arêas, 2013; Mollakhalili‐Meybodi et al., 2021; Ziobro et al., 2013). ITFs are water‐soluble storage polysaccharides composed of fructosyl units attached by β‐(2–1) bonds (polymerization degree of 2–60) with a glucose unit reducing end. They are potentially beneficial to consumers as a rich source of dietary fiber (Luo et al., 2017).

Fermentation process as one of the basic steps in making bread with the aim of improving the nutritional value and quality is generally done using yeast or sourdough (Capriles & Arêas, 2013). While only *Saccharomyces cerevisiae* is involved in processes done by yeast, the native microflora of flour which is generally a mixture of lactic acid bacteria and yeasts are characterized in sourdough (Belz et al., 2012). Considering the health characteristics of inulin and the dependence of quality parameters of gluten‐free bread on inulin structure, the ratio of combined flours, and the type of fermentation used, this study is aimed to introduce the optimal formulation of gluten‐free bread enriched with inulin, considering the effect of acorn flour replacement degree and type of fermentation on its technological characteristics.

## 2 MATERIALS AND METHODS

### 2.1 Materials

Commercial rice flour (6.95 ± 0.01% W/W moisture, 0.39 ± 0.05% W/W ash, 9.33 ± 0.01% W/W protein, and 1.32 ± 0.03% W/W fat) was purchased from Golha Company. Acorn flour (5.95% W/W moisture, 6.82 ± 0.39% W/W ash, 5.40 ± 0.03% W/W protein, and 8.47 ± 0.13% W/W fat) was provided from local market. Long‐chain inulin (Frutafit TEX, inulin content ≥99.5% DP ≥23) was kindly donated from Akbarieh Company. The other constituents of gluten‐free breads (salt, sugar, oil, and active dry yeast) were purchased from local supermarkets. The required sodium hydroxide was also purchased from Merck Company for titration.

### 2.2 Bread making process

**FIGURE 1:**
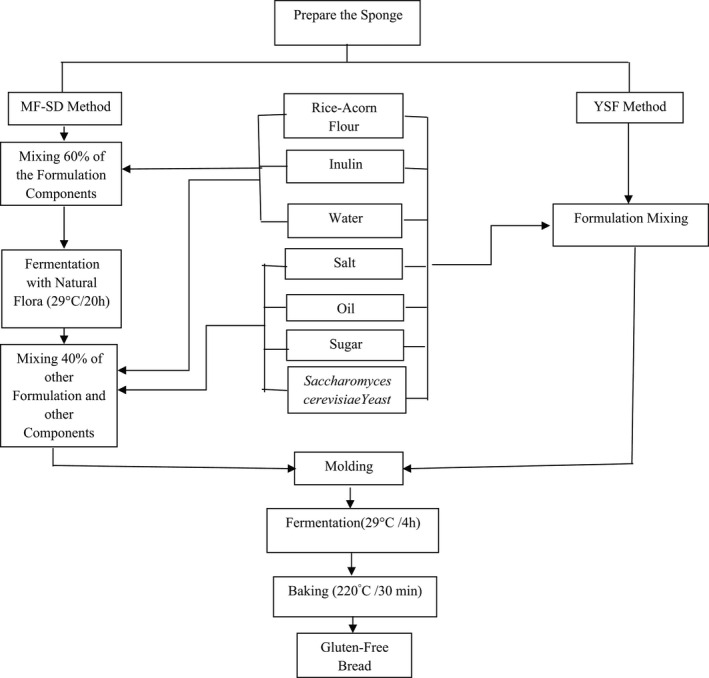
The schematic diagram of gluten‐free bread preparation steps

The influence of acorn flour replacement was investigated at three levels (10, 30, 50% W/W). Long‐chain inulin was replaced at 10% W/W in flour blends (acorn–rice flour ratio at 10:90, 30:70, and 50:50). The water absorption in each flour blend containing 10% W/W long‐chain inulin was determined by rheometer (Anton Paar MCR301, GmbH) adjustment to achieve an optimum dough consistency. In order to investigate the influence of fermentation types (yeast starter fermentation [Y] or mixed fermentation based on sourdough [MF‐SD]), the formulations were prepared as follow. The schematic diagram of gluten‐free bread preparation steps is declared in Figure 1.

#### 2.2.1 Yeast starter fermentation (YSF)

Straight dough method was used to prepare YSF dough. Regarding, acorn: rice blend flour (10:90, 30:70, and 50:50% W/W), containing 10% W/W long‐chain inulin were mixed by sugar, salt, oil, and active dry yeast at 0.5, 1, 3, and 2.2% W/W, respectively. The blends were then mixed with potable water at amounts as determined by rheometer. The mixture is shaped into dough then putted in baking pans and incubated for 4 h at temperature adjusted to 29±0.5℃. A piece of samples (30 g) was kept for rheological parameter determination and the other used for baking. The baking process is done in a convection oven (Model PFB‐2, Duke manufacturing Company) for 30 min at 220 ± 0.5℃ (Gamel et al., 2015).

#### 2.2.2 Mixed fermentation based on sourdough (MF‐SD)

**FIGURE 2:**
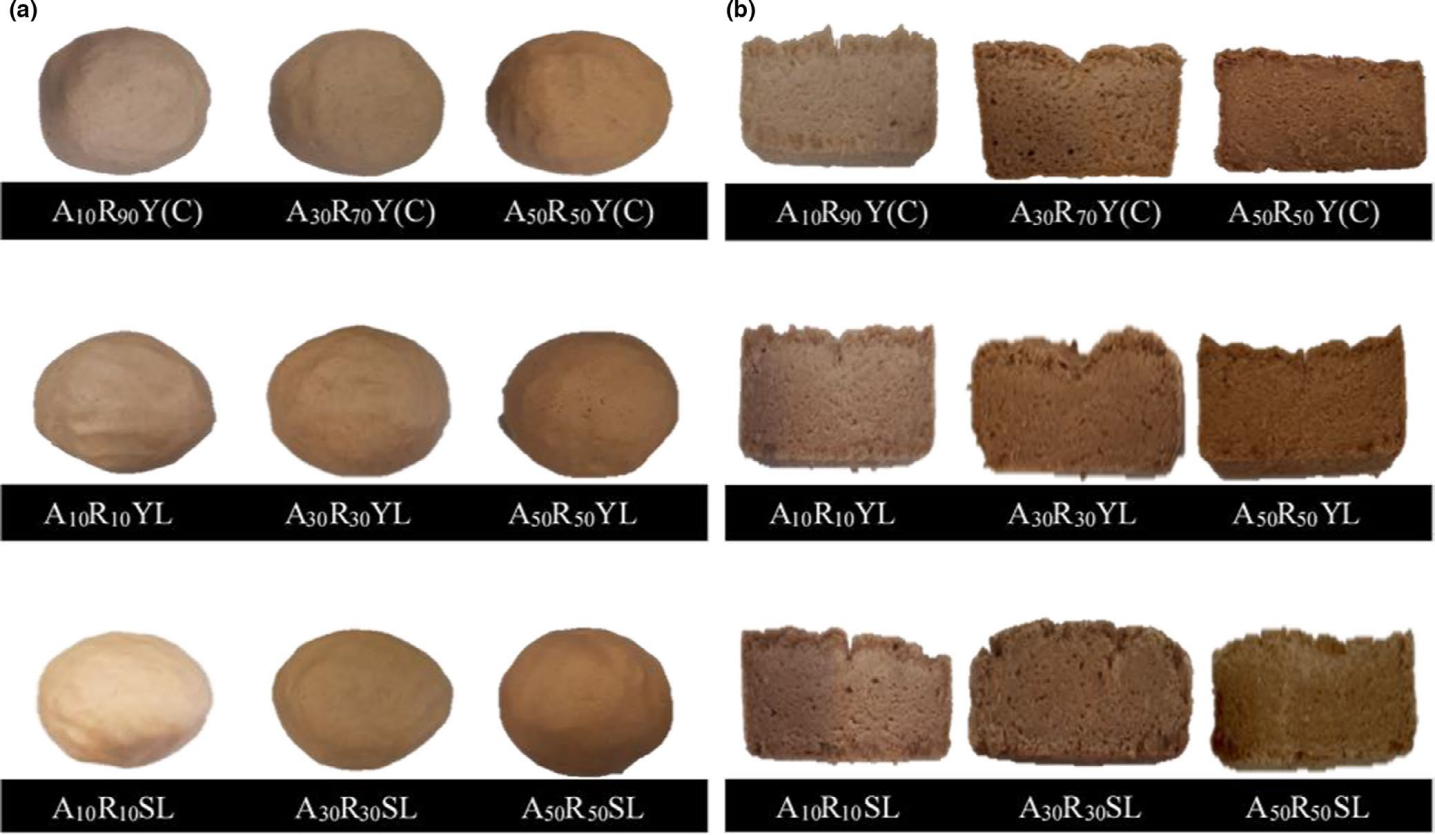
(a) The appearance of the dough samples; (b) Cross section of bread samples prepared in the present study

Mixed fermentation based on sourdough based breads were prepared on the basis of the sponge and dough method with a proportion of 60:40 (sponge: dough) (Gamel et al., 2015). The sponge was prepared by 60% W/W acorn: rice flour (10:90, 30:70, and 50:50) containing 10% W/W inulin and 60% W/W water as determined by the rheometer. After mixing, the batter is putted in a bowl and held for 20 h in the fermentation cabinet with a temperature of 29 ± 0.5℃. The other 40% W/W acorn: rice flour containing 10% W/W inulin and water were mixed by sugar, salt, oil, and active dry yeast at 0.5, 1, 3, and 2.2% W/W as dough and mixed by batter. The mixture was then incubated for 4 h at 29 ± 0.5℃. The dough preparation and baking process were done similar to abovementioned processes (Gamel et al., 2015). Rheological characteristics were determined on dough before baking. The appearance of the dough samples is shown in Figure 2a.

### 2.3 Technological characteristics

#### 2.3.1 Rheological characteristics of dough

Oscillatory shear characteristics of dough samples were determined by controlled shear/stress rheometer (Anton Paar MCR301, GmbH) using parallel plate geometry at 30℃. Dough sample pieces were rested for 5 min. Strain sweep test was carried out firstly to determine the linear viscoelastic region. Since results indicated the linear behavior of dough at strain lower than 0.1%, frequency sweep test was done at a range of 1–100 Hz at constant strain of 0.01%. The damping factor (tan *δ*) and complex modulus (G*) were determined as follow (Upadhyay et al., 2012):

$$\tan\;\delta =\frac{G^{\prime \prime }}{G^{\prime }}$$

$$G^{*}=\sqrt{G^{\prime 2}+G^{\prime \prime 2}}$$

#### 2.3.2 Physicochemical characteristics

##### Specific volume

The loaf volume of breads was determined by rapeseed displacement method, and its specific volume was calculated from loaf volume divided by weight (AACC Method 10‐05.01), nearly 1 h after leaving the oven (Gerrard et al., 2003).

##### pH and TTA

About 5 g of bread sample was mixed with 50 ml of distilled water, and the pH value was determined by pH meter after homogenizing (SANA SL‐901). Then, total titratable acidity (TTA) was determined by titration with 0.1 N sodium hydroxide (Katina, Salmenkallio‐Marttila, et al., 2006).

##### Moisture content

The moisture content of the bread samples was determined using the oven drying method (AACC44‐16). Samples were placed in an air oven set at 105 ± 0.05℃, and the drying process was continued until the mass change of two weighing intervals of 15 min was less than 0.1% W/W. The moisture content was calculated as follow (Lu et al., 2014).

$$\text{Moisture}\;\text{content}=\frac{M\;\text{sample}‐M\;\text{after drying}}{M\;\text{sample}}\times 100\%$$

where the M sample and M after drying are the mass (g) of bread samples before and after drying, respectively.

#### 2.3.3 Textural characteristics of breads

Instrumental texture parameters were calculated using texture profile analyzer (TA20., KOOPA) according to a modified AACC Approved Method 74‐09 (2000). A piece of the crumb (20 × 20 × 25 mm) was pressed to 50% of its original height at speed of 1 mm/s with a 43 mm cylinder probe using 5 kg loading cell. The analysis was carried out in six replicates at 25 ± 3℃ on the bread slices. Hardness, cohesiveness, springiness, and chewiness of the crumb were calculated using Texture profile analysis curves (Katina, Heiniö, et al., 2006). Cross section of bread samples prepared in the present study is presented in Figure 2b.

#### 2.3.4 Crust and crumb color determination

In order to determine the color of the specimens, the Hunter Lab instruments (D25‐9000 made in Germany) were used. In this regard, the crust and crumb color of each bread pieces were determined. Results were reported as L* (brightness‐zero: black and 100: white), a* (green: negative and red: positive), and b* (blue: negative and yellow: positive) indices. White index (WI) was obtained based on the following formula (Sardabi et al., 2021).

$$\text{WI}=100‐\sqrt{{\left(100‐L^{*}\right)}^{2}+{\left(a^{*}\right)}^{2}+{\left(b^{*}\right)}^{2}}$$

#### 2.3.5 Sensory evaluation

Samples prepared at this study were sensory investigated using nine‐point hedonic scale with 1 indicative of very unpleasant, 5 acceptable, and 9 extremely pleasant samples. Assessment was done according to flavor, color, texture, and the overall acceptability by 30 semitrained panelists from students and staff (male: female ratio of 50:50 and 18–58 years old) from school of public health, Shahid Sadoughi University of Medical Science. The panelist's good health and willingness to be participated has been considered. For sensory evaluation, the encoded bread sample slices were served randomly and plain water was served before and between testing (Menon et al., 2015).

### 2.4 Statistical analysis

In this study, descriptive statistics using mean and standard deviation were used to describe quantitative features. In order to obtain the highest accuracy in the results of statistical analysis, the relevant tests were performed in three replications. Data analysis was performed using SPSS V.21 software. Finding a significant difference between the mean data of the applied treatments was done using two‐way ANOVA in which acorn flour substitution level and fermentation are the independent variables. The homogenous groups were determined by Tukey's test at a significance level of 0.05. Nonparametric tests (Kruskal–Wallis) were used for sensory evaluation.

## 3 RESULTS AND DISCUSSION

### 3.1 Dough rheology characterization

**FIGURE 3:**
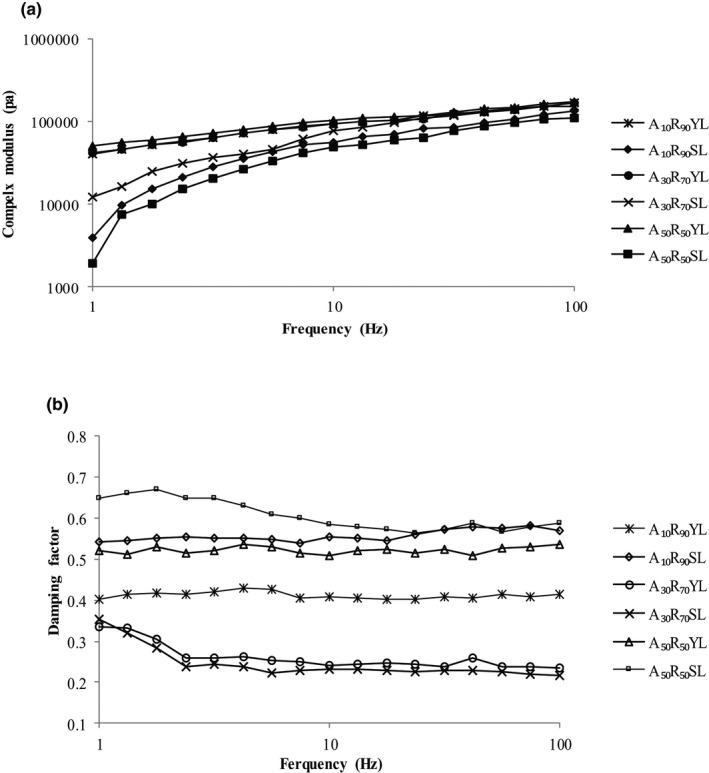
Frequency sweep curves of the experimental GF dough samples (*curves are the mean of at least two replicates*) ([a] and [b] are complex modulus [|G*|] and damping factor [tan δ], respectively)

The viscoelastic characteristics of gluten‐free dough samples have been determined to investigate the influence of changes in formulations (Witczak et al., 2017). The frequency sweep test has been done to determine the manner in which the viscose and elastic behavior of material change with the rate of stress or strain, as the amplitude of the signal is kept constant. The frequency sweep curves of experimental gluten‐free dough samples are represented in Figure 3a,b. For all formulations in the whole range of angular frequencies, the storage modulus was greater than loss modulus (G′ > G″), representing the formation of elastic‐like behavior of the gluten‐free dough as demonstrated by Upadhyay et al. (2012).

The highest damping factor and lowest complex modulus has been observed in A_50_R_50_SL demonstrating the remarkable enhancement of viscose modulus via inclusion of acorn flour at 50% W/W and MF‐SD fermentation type. The decrease in complex modulus is indicative of a decrease in the viscous and elastic characteristic and consequently the overall dough strength (Marcoa & Rosell, 2008). The increase in damping factor through increasing the level of acorn flour suggests the decrease in elastic characteristics of dough (Lahiji et al., 2013). The structure weakening impact and consequently elasticity reduction of gluten‐free dough via increasing the level of acorn flour inclusion has been previously stated by Beltrão Martins, Gouvinhas, et al. (2020) and Beltrão Martins, Nunes, et al. (2020). The complex modulus in all samples has been increased via increasing the angular frequency. In this among, the lowest frequency dependency has been observed in A_30_R_70_SL, indicating the formation of strong elastic structure (Demirkesen et al., 2010a).

Considering the influence of fermentation type, it has been declared that using MF‐SD resulted in decreasing both the elastic and viscous modulus (data not shown). It seems that protein degradation proposed by mixed fermentation based on sourdough has weaken the dough strength and consequently the complex modulus (Clarke et al., 2004; Sandra et al., 2012).

An increase in damping factor was observed at all acorn flour inclusion level. The increase observed in damping factor indicates that the elastic component decreases to a greater extent which is consistent with the findings of the Galle et al. (2012). However, considering the lowest damping factor observed in A_30_R_70_SL, it seems that the exopolysaccharides produced by lactic acid bacteria in MF‐SD can have a synergistic effect with long‐chain inulin by creating a gel‐like structure to increase the elastic component and thus reduce the damping factor (Beltrão Martins, Gouvinhas, et al., 2020; Beltrão Martins, Nunes, et al., 2020; Galle et al., 2012; Juszczak et al., 2012).

### 3.2 Physicochemical characteristics of gluten‐free breads

Gluten‐free bread samples were prepared with 10, 30, and 50% W/W acorn flour replacement and 10% W/W inulin incorporation in the presence of two fermentation methods (yeast starter and MF‐SD). The appearance and cross section of prepared samples are presented in Figure 2.

#### 3.2.1 Moisture content

**FIGURE 4:**
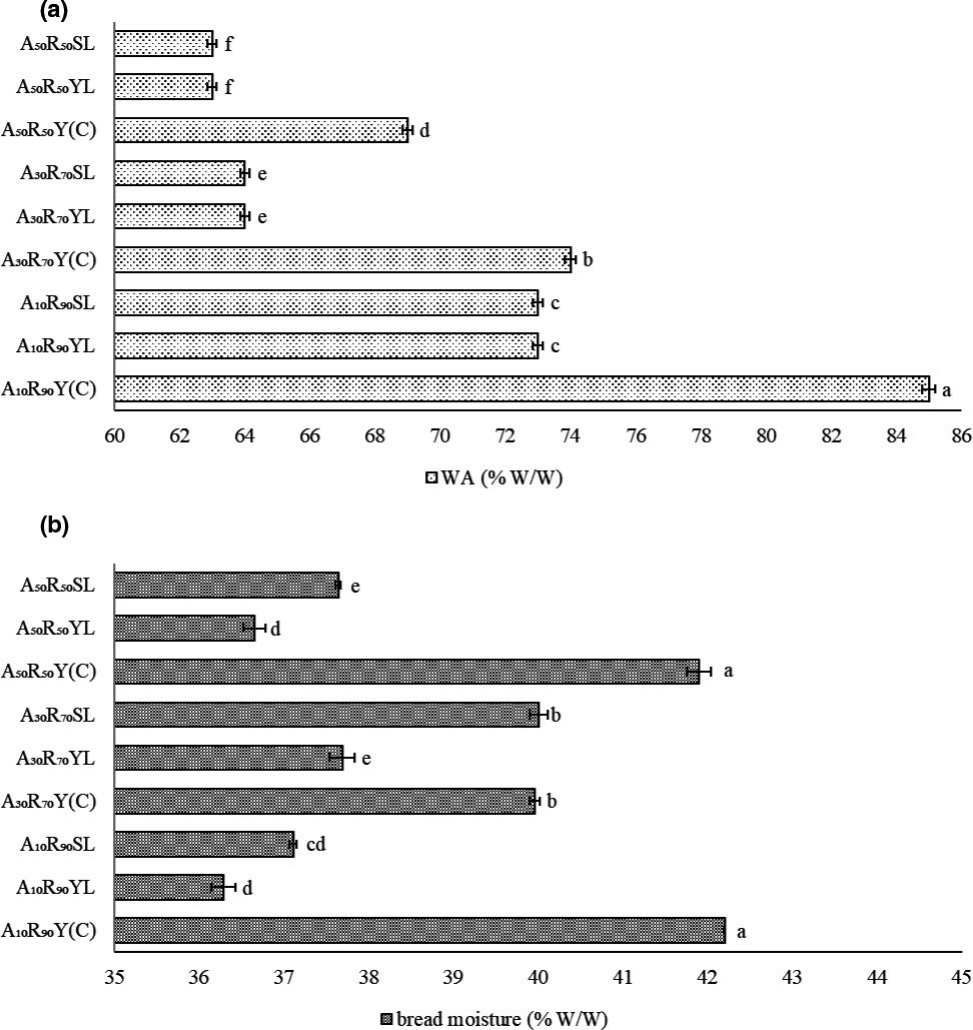
(a) and (b) are water absorption (% W/W) of the dough and final moisture (% W/W) of the gluten‐free breads. The different lowercase letters mean the significant difference (*p* ≤ .05) in water absorption (WA) and bread moisture

As declared in Figure 4, the moisture content has significantly been decreased by long‐chain inulin incorporation and increasing the ratio of acorn flour from 10% to 50% W/W (*p* ≤ .05). This finding showed that long‐chain inulin fortification of gluten‐free bread could produce a more shelf‐stable bread due to its lower moisture content (Taghdir et al., 2017). Using MF‐SD has also seen to enhance the moisture content which is supposed to be induced by its higher enzymatic activity and consequently higher hydrophilic characteristics of hydroxyl groups (Escrivá & Martínez‐Anaya, 2000).

Considering both the water absorption and moisture content, it has shown that they have changed similarly. In other words, the lowest and highest moisture content has been observed in samples with lowest and highest water absorption content, respectively (A_50_R_50_YL and A_10_R_90_Y(C)). However, reverse trend has been observed in A_30_R_70_SL and A_10_R_90_YL. The increase in moisture content of A_30_R_70_SL sample despite its lower water absorption ratio is supposed to be induced by the synergistic impacts of long‐chain inulin and exopolysaccharides produced by lactic acid bacteria to form gel with lower gelatinization temperature. The formation of gel will result in space restriction which reduced the cross linking of starch molecules to form an ordered structure, prevented the moisture migration in the gel, and consequently enhanced its water holding capacity. The increase in moisture content induced by MF‐SD fermentation type is dependent to the level of acorn flour incorporation. In other words, it was 2.25, 6.15, and 2.70% W/W at 10, 30, and 50% W/W acorn flour substitution, respectively. As acorn flour is considered as a rich source of dietary fiber, its enhancement up to 30% W/W facilitated the formation of gels. Increasing the acorn flour incorporation level to 50% W/W seems to decrease the potential of gel formation which is attributed to the dominance of gel inhibitory impacts of acorn flour as reported by Paciulli et al. (2016).

#### 3.2.2 Acidification analysis

**FIGURE 5:**
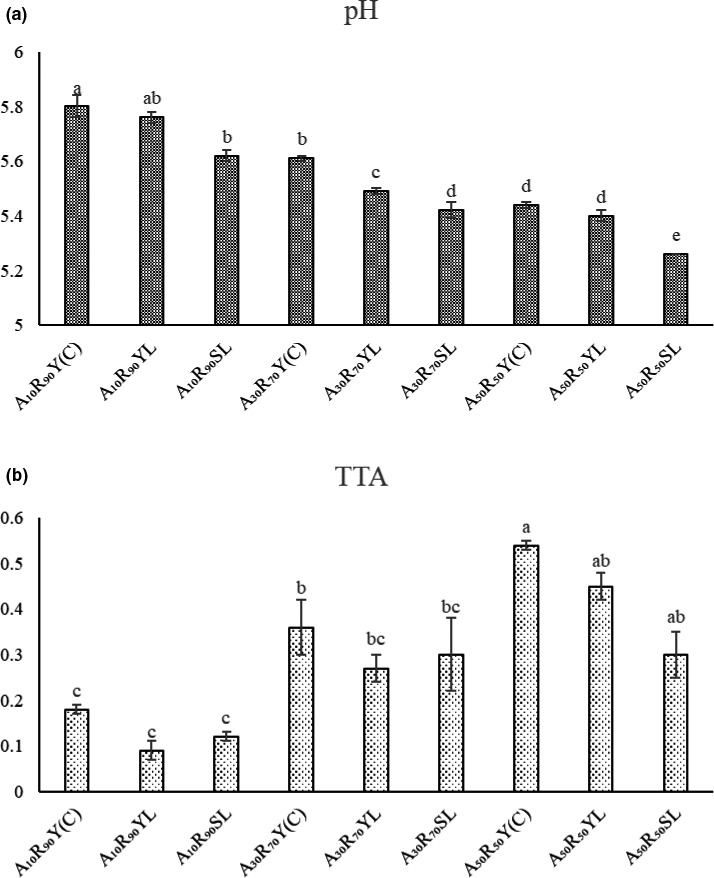
(a) and (b) are pH and total titratable acidity (TTA) of gluten‐free bread samples. The different lowercase letters mean the significant difference (*p* ≤ .05) in TTA and pH

Acidification process (as determined by pH and TTA) is considered as the key parameter in techno‐functional determination of bread. The acidification process in gluten‐free bread preparation depending on the type of fermentation and level of acorn flour used is presented in Figure 5. Incorporation of MF‐SD in dough formulation decreased the pH and increased the TTA in bread samples depending on the level of acorn flour. In other words, the pH values had been decreased by increasing the level of acorn flour incorporation. The acidification in bread is influenced by the substrate, microbiota, and fermentation condition (Purabdolah et al., 2020). It has been reported that substrate constituent (protein, carbohydrate, and ash content) and their buffering capacity are provital in determining the pH and TTA content of final product.

### 3.3 Gluten‐free bread structural characteristics

The specific volume as an indicator of gas retention capacity of dough and its expansion ratio during the baking process (44) is reported in the range of 1.14–1.49 (cm^3^ g^‐1^) (Table 1). The highest specific volume is found in A_30_R_70_SL and A_30_R_70_Y(C) with no significant difference (*p* > .05). A gradual decrease has been observed via inulin inclusion in samples fermented by yeast starter. Korus et al. (2006) observed a similar result in which the volume of gluten‐free bread has been decreased by the addition of 3% W/W inulin (45). Dietary fiber has been reported to negatively influence the bread quality by decreasing its specific volume induced by its lower gas retention capacity (Jagelaviciute & Cizeikiene, 2021).

**TABLE 1:** Texture profile analysis of gluten‐free breads

Trial	Properties				
	Specific volume (cm^3^ g^−1^)	Hardness (*N*)	Springiness (mm)	Cohesiveness (−)	Chewiness (mJ)
A_10_R_90_Y(C)	1.36 ± 0.01^ab^	71.17 ± 0.85^e^	9.19 ± 0.25^d^	0.45 ± 0.01^a^	207.70 ± 4.11^a^
A_10_R_90_YL	1.25 ± 0.05^b^	106.09 ± 0.88^b^	6.56 ± 0.30^f^	0.24 ± 0.02^c^	109.74 ± 3.07^b^
A_10_R_90_SL	1.20 ± 0.07^b^	75.67 ± 0.78^d^	11.50 ± 0.18^b^	0.28 ± 0.01^bc^	65.72 ± 4.05^c^
A_30_R_70_Y(C)	1.49 ± 0.03^a^	30.03 ± 0.93^h^	9.28 ± 0.29^d^	0.41 ± 0.03^a^	59.86 ± 2.50^c^
A_30_R_70_YL	1.14 ± 0.07^b^	60.20 ± 0.87^f^	10.01 ± 0.24^c^	0.27 ± 0.03^c^	54.35 ± 2.90^c^
A_30_R_70_SL	1.47 ± 0.04^a^	40.97 ± 0.87^g^	12.50 ± 0.21^a^	0.40 ± 0.02^a^	40.92 ± 3.40^c^
A_50_R_50_Y(C)	1.16 ± 0.03^b^	59.66 ± 0.94^f^	8.90 ± 0.30^d^	0.42 ± 0.01^a^	114.62 ± 4.32^b^
A_50_R_50_YL	1.14 ± 0.04^b^	99.18 ± 0.89^c^	8.90 ± 0.23^d^	0.34 ± 0.02^b^	162.45 ± 3.06^b^
A_50_R_50_SL	1.25 ± 0.07^b^	131.0 ± 0.93^a^	7.40 ± 0.35^e^	0.24 ± 0.03^c^	180.74 ± 1.98^b^

Note

Data are reported as average ± standard deviation. The values with the different lowercase letter in each column mean the significant difference (*p* ≤ .05). A_a_ = level of acorn flour (A_10_: 10% W/W, A_30_: 30% W/W, A_50_: 50% W/W), R_b_ = level of rice flour (R_90_: 90% W/W, R_70_: 70% W/W, R_50_: 50% W/W), X = type of fermentation (Y = yeast starter, S = mixed fermentation based on sourdough), I = long‐chain inulin (L). Control samples are shown with A_a_R_b_Y (C).

Regarding the influence of sourdough on specific volume of gluten‐free breads, contradictory results had been reported (Moroni et al., 2009; Steffolani et al., 2014; Wolter et al., 2014). In this study, the influence of fermentation type is only significant at formulations containing 30% W/W acorn flour. In other words, using MF‐SD increased its specific volume significantly with no significant difference with the control sample (*p* > .05). The higher ability of MF‐SD to produce gases, which can be due to the synergistic effects of lactic acid bacteria on the metabolic activity of *Saccharomyces cerevisiae*, leads to more release of carbon dioxide gas (Katina, Heiniö, et al., 2006; Katina, Salmenkallio‐Marttila, et al., 2006). In addition, the synergistic effects of exopolysaccharides produced in mixed fermentation based on sourdough and long‐chain inulin in creating a favorable structure capable of holding gas can increase the specific volume (Lynch et al., 2018).

A change in specific volume of bread is generally accompanied by hardness change. In this study, increasing the ratio of acorn flour from 10% W/W to 30% W/W decreased the hardness significantly (*p* ≤ .05). However, it has been increased by increasing the ratio of acorn flour incorporation from 30% W/W to 50% W/W (*p* ≤ .05). The lowest hardness in A_30_R_70_SL is coinciding with its higher specific volume compared to other samples. It seems to be induced through the effect of formulation on the specific volume (Demirkesen et al., 2010b; Sheikholeslami et al., 2019). Incorporation of inulin increased the hardness in all formulations through decreasing the specific volume. The influence of fermentation type on the hardness is affected by the ratio of acorn–rice flour. While using mixed fermentation based on sourdough decreased the hardness in A_10_R_90_ and A_30_R_70_ samples, increased it in samples containing 50% W/W acorn flour (A_50_R_50_SL). The increased hardness of A_50_R_50_SL is attributed to its higher moisture content which is similar to results obtained by Tsatsaragkou et al. (2016).

Cohesiveness as an indicator of the internal bands strength is influenced by dough formulation. Despite no significant different (*p* > .05) via inclusion of acorn flour at different ratio, incorporation of inulin at 10% W/W decreased the cohesiveness differently. The quantity of cohesiveness parameter is reported to be dependent to the formulation structure (Wahyono et al., 2016). Considering the influence of inulin to weaken the structure (Liu et al., 2016), lower decrease is observed in lower structured formulation (A_50_R_50_Y(C)). Results also indicated that using mixed fermentation based on sourdough can compensate the cohesiveness decrease induced by inulin incorporation which is attributed to its potential to produce exopolysaccharides and its synergistic impact to provide a gel‐like structure (Beltrão Martins, Gouvinhas, et al., 2020; Beltrão Martins, Nunes, et al., 2020; Galle et al., 2012; Juszczak et al., 2012). This impact is reversely observed in A_50_R_50_ samples which using MF‐SD decreased their cohesiveness compared to yeast starter fermented ones (*p* ≤ .05). As proteins play an important role in structural cohesiveness, the apparent decrease in protein content (7.36% W/W) (at high ratios of acorn flour) and the hydrolysis facilitated by of MF‐SD are considered as the main reasons in this regard (Loponen et al., 2004).

Springiness indicates the rate of return of a deformed material to its normal state after removal of the deformation force (Onyango et al., 2010). The highest springiness contents found in A_30_R_70_SL have revealed the increase in its tensile strength which is in accordance with rheological properties finding. Chewiness as an indicator of the energy needs to convert a solid food into a digestible form has shown a trend similar to the hardness of the samples which has been previously stated by Abdelghafor et al. (2011).

### 3.4 Color analysis

The impact of inulin incorporation and fermentation type on the crumb and crust color of acorn: rice (different ratio) gluten‐free bread has been investigated and presented in Table 2. Color parameters are expressed as CIE L*, a*, and b* which are indicators of lightness, green (−)/red (+), and blue (−)/yellow (+), respectively. While the crust is the surface brown layer of bread, the crumb is the white spongy structure under it (Jusoh et al., 2009). The lightest bread crust is observed in A_10_R_90_YL which is attributed to its lower moisture content as reported by Masure et al. (2016). The color characteristics of bread crust are mainly influenced by the Millard reaction (Conforti & Davis, 2006). Results indicated that increasing the ratio of acorn flour incorporation increased the crust lightness which is attributed to its lower protein content and consequently lower Millard reaction occurrence. The formation of pigments through Millard reaction will also increase the redness and yellowness as presented by the lower a* and b* in samples containing higher ratio of acorn flour incorporation (Paciulli et al., 2016). Reducing sugars and proteins can react through the Millard reaction at high temperatures of baking process will promote the darkening of the final products. Therefore, the decrease in brightness and white index (WI) can be attributed to Millard reaction (Rayan et al., 2018).

**TABLE 2:** Color parameters (L*, a*, b*, and white index) of crust and crumb of gluten‐free bread samples

Trial	Crust properties				Crumb properties			
	L*	a*	b*	WI	L*	a*	b*	WI
A_10_R_90_Y(C)	84.05 ± 0.76^d^	10.31 ± 0.00^c^	23.90 ± 0.17^c^	69.47 ± 0.13^c^	90.78 ± 0.16^a^	7.01 ± 0.01^f^	26.30 ± 0.15^c^	71.26 ± 0.08^b^
A_10_R_90_YL	89.29 ± 0.25^a^	8.95 ± 0.14^e^	26.78 ± 0.09^ab^	69.80 ± 0.10^b^	87.45 ± 0.17^e^	10.14 ± 0.06^b^	25.45 ± 0.11^e^	69.87 ± 0.14^cd^
A_10_R_90_SL	87.17 ± 0.05^b^	11.41 ± 0.16^b^	27.16 ± 0.38^a^	67.87 ± 0.11^e^	87.95 ± 0.15^d^	10.20 ± 0.09^b^	25.81 ± 0.05^d^	69.74 ± 0.11^d^
A_30_R_70_Y(C)	85.68 ± 0.14^c^	11.61 ± 0.01^b^	25.83 ± 0.33^b^	68.27 ± 0.06^d^	89.66 ± 0.11^b^	8.55 ± 0.01^e^	26.52 ± 0.06^c^	70.28 ± 0.14^c^
A_30_R_70_YL	85.12 ± 0.43^cd^	12.02 ± 0.09^a^	25.85 ± 0.31^b^	67.84 ± 0.13^e^	88.36 ± 0.12^d^	9.74 ± 0.06^c^	26.91 ± 0.05^b^	69.10 ± 0.10^e^
A_30_R_70_SL	84.95 ± 0.15^cd^	11.53 ± 0.06^b^	25.73 ± 0.19^b^	68.25 ± 0.06^d^	84.73 ± 0.07^f^	11.31 ± 0.02^a^	24.85 ± 0.06^f^	68.72 ± 0.10^e^
A_50_R_50_Y(C)	87.30 ± 0.07^b^	11.43 ± 0.15^b^	27.20 ± 0.30^a^	68.27 ± 0.13^d^	88.98 ± 0.14^cd^	9.57 ± 0.03^c^	27.28 ± 0.03^a^	69.06 ± 0.06^e^
A_50_R_50_YL	87.18 ± 0.65^b^	10.74 ± 0.11^d^	23.54 ± 0.03^c^	71.12 ± 0.09^a^	88.68 ± 0.07^c^	9.67 ± 0.07^c^	24.38 ± 0.04^f^	71.43 ± 0.14^ab^
A_50_R_50_SL	87.39 ± 0.04^b^	10.52 ± 0.04^cd^	24.00 ± 0.27^c^	70.92 ± 0.07^a^	89.38 ± 0.17^bc^	9.08 ± 0.09^d^	24.55 ± 0.15^f^	71.75 ± 0.09^a^

Note

Data are reported as average ± standard deviation. The values with the different lowercase letters in each column mean the significant difference (*p* ≤ .05). A_a_ = level of acorn flour (A_10_: 10% W/W, A_30_: 30% W/W, A_50_: 50% W/W), R_b_ = level of rice flour (R_90_: 90% W/W, R_70_: 70% W/W, R_50_: 50% W/W), X = type of fermentation (Y = yeast starter, S = mixed fermentation based on sourdough), I = long‐chain inulin (L). Control samples are shown with A_a_R_b_Y (C).

The crumb color is generally determined by the formulation ingredients and its specific volume and hardness (Conforti & Davis, 2006). As demonstrated in Table 2, increasing the level of acorn flour incorporation decreased the lightness and increased the yellowness and redness significantly (*p* ≤ .05) which was similar to finding of Beltrão Martins, Gouvinhas, et al. (2020) and Beltrão Martins, Nunes, et al. (2020). Generally the colorimetric changes induced by acorn flour incorporation are generally preferred by the consumers regarding the unpleasant pale color of gluten‐free products.

The lowest crumb lightness has been observed in A_30_R_70_SL sample containing 30% W/W acorn flour and fermented by mixed fermentation based on sourdough. As the highest specific volume is also observed at A_30_R_70_SL, it seems that its increased air bubbles incorporation and light scattering resulted in a decrease in L* value of the product (Rinaldi et al., 2015). The A_30_R_70_SL sample showed the lowest crumb white index too. The increase in specific volume was associated with a decrease in L* and consequently the crumb white index. It seems that in addition to the color of the formulations, the specific volume is also effective in determination of crumb white index. In the study of Hsieh et al., It was observed that L* is directly related to white index (Hsieh et al., 2017).

### 3.5 Sensory analysis

The sensory profile of breads prepared in this study is presented in Table 3. In this regard, flavor, texture, color, and overall acceptability have been evaluated in formulations on the basis of the level of acorn flour, inulin incorporation, and fermentation type. No significant difference has been observed by the consumers via inclusion of inulin (*p* > .05).

**TABLE 3:** Sensory evaluation of gluten‐free bread prepared with different formula

Trial	Properties			
	Flavor	Color	Texture	Overall acceptability
A_10_R_90_Y(C)	6.33 ± 0.10^b^	6.44 ± 0.10^b^	7.11 ± 0.04^bc^	6.66 ± 0.05^c^
A_10_R_90_YL	6.33 ± 0.30^b^	6.44 ± 0.30^b^	7.11 ± 0.18^bc^	6.33 ± 0.11^c^
A_10_R_90_SL	6.33 ± 0.25^b^	6.44 ± 0.39^b^	6.88 ± 0.05^c^	6.22 ± 0.18^c^
A_30_R_70_Y(C)	7.44 ± 0.15^a^	7.66 ± 0.29^a^	7.66 ± 0.14^b^	7.88 ± 0.30^b^
A_30_R_70_YL	7.44 ± 0.32^a^	7.22 ± 0.40^a^	8.33 ± 0.09^a^	7.66 ± 0.18^b^
A_30_R_70_SL	7.44 ± 0.22^a^	7.44 ± 0.15^a^	8.66 ± 0.18^a^	8.22 ± 0.07^a^
A_50_R_50_Y(C)	5.66 ± 0.40^c^	5.33 ± 0.27^c^	6.66 ± 0.12^c^	5.66 ± 0.06^d^
A_50_R_50_YL	5.66 ± 0.19^c^	5.44 ± 0.40^c^	6.11 ± 0.17^d^	5.44 ± 0.20^de^
A_50_R_50_SL	5.66 ± 0.10^c^	5.33 ± 0.21^c^	7.66 ± 0.16^b^	5.33 ± 0.15^e^

Note

Data are reported as average ± standard deviation. The values with the different lowercase letter in each column mean the significant difference (*p* ≤ .05). A_a_ = level of acorn flour (A_10_: 10% W/W, A_30_: 30% W/W, A_50_: 50% W/W), R_b_ = level of rice flour (R_90_: 90% W/W, R_70_: 70% W/W, R_50_: 50% W/W), X = type of fermentation (Y = yeast starter, S = mixed fermentation based on sourdough), I = long‐chain inulin (L). Control samples are shown with A_a_R_b_Y (C).

Considering the overall acceptability perceived by the consumers, the highest acceptability has been observed in A_30_R_70_SL containing 30% W/W acorn flour and 10% W/W inulin in the presence of MF‐SD. As no significant difference has been observed in flavor and color assessment resulting the type of fermentation process, its impact is evident in textural perception and overall acceptability. Considering the texture parameter, it seems that using MF‐SD which negatively influences the textural perception at formulations containing 10% W/W acorn flour improve the texture at those having higher quantity of acorn flour. The highest acceptability in texture perception is achieved by A_30_R_70_SL which is attributed to synergistic effect of gel formation by exopolysaccharides produced by MF‐SD and long‐chain inulin (Katina, Heiniö, et al., 2006; Katina, Salmenkallio‐Marttila, et al., 2006; Sirbu & Arghire, 2017). Improving observed in flavor and color perception at 30% W/W acorn flour inclusion seems to be induced to improving the pale color and tasteless characteristics of the rice flour via inclusion of 30% W/W acorn flour (Demirkesen et al., 2010a). However, its higher inclusion level had adversely changed the color, flavor, texture, and consequently the overall acceptability. In this regard, optimizing the formulation needs to be done.

The correlation analysis of sensory perception and instrumental measurement through the current study has revealed that the specific volume and cohesiveness were positively correlated with sensory texture scores (*r* = .458, .411, respectively, *p* ≤ .05). On the other hand, chewiness was negatively correlated with the panelists' scores for texture (*r* = −.602, *p* ≤ .05). Moreover, the scores for color were significantly and negatively correlated with crumb lightness and crumb and crust white index (*r* = −.493, −.541, −.405, respectively, *p* ≤ .05).

## 4 CONCLUSIONS

Acorn flour is potentially influencive in improving the technological characteristics of gluten‐free bread. However its optimization regarding the fermentation type and other constituents needs to be obtained. Considering results obtained at this study, using mixed fermentation based on sourdough and long‐chain inulin at 10% W/W provided the structure able to restore gases through baking process at formulations containing acorn flour at 30% W/W which seems to be induced by its ability to form gel. Acorn flour substitution at 50% W/W had adversely influenced the technological characteristics of final product. From consumer's perspective, the acorn flour substitution level at 30% W/W is preferred regarding its potential role to improve the unpleasant pale color of rice‐based gluten‐free products.

## CONFLICT OF INTEREST

The authors declare that they have no conflict of interest.

## AUTHOR CONTRIBUTIONS

**Ameneh Shiri:** Investigation (equal); Project administration (equal); Writing‐original draft (equal); Writing‐review & editing (equal). **Mohammad Hassan Ehrampoush:** Investigation (equal); Writing‐original draft (equal); Writing‐review & editing (equal). **Seyed Ali Yasini Ardakani:** Investigation (equal); Writing‐original draft (equal); Writing‐review & editing (equal). **Farimah Shamsi:** Investigation (equal); Writing‐original draft (equal); Writing‐review & editing (equal). **Neda Mollakhalili‐Meybodi:** Project administration (equal); Visualization (equal); Writing‐original draft (equal); Writing‐review & editing (equal).

## ETHICAL APPROVAL

This study does not involve any human or animal testing. Written informed consent was obtained from all study participants. This study was approved by the Institutional Review Board of School of public health, Shahid Sadoughi University of Medical Sciences. Approval ID: IR.SSU.SPH.REC.1399.014.

## Data Availability

Data will be available within the article.
